# Role of ROBO4 Signalling in Developmental and Pathological Angiogenesis

**DOI:** 10.1155/2014/683025

**Published:** 2014-02-06

**Authors:** Suresh Singh Yadav, Gopeshwar Narayan

**Affiliations:** Cancer Genetics Laboratory, Department of Molecular and Human Genetics, Banaras Hindu University, Varanasi 221 005, India

## Abstract

Transmembrane roundabout receptor family members (ROBO1–ROBO4) principally orchestrate the neuronal guidance mechanism of the nervous system. Secreted glycoprotein SLITs are the most appreciated ligands for ROBOs. Recently identified ROBO4 is the key mediator of SLIT-ROBO mediated developmental and pathological angiogenesis. Although SLIT2 has been shown to interact with ROBO4 as ligand, it remains an open question whether this protein is the physiologic partner of ROBO4. The purpose of this review is to summarise how reliable SLIT2 as ligand for ROBO4 is, if not what the other possible mechanisms demonstrated till date for ROBO4 mediated developmental and pathological angiogenesis are. We conclude that ROBO4 is expressed specially in vascular endothelial cells and maintains the vascular integrity via either SLIT2 dependent or SLIT2 independent manner. On the contrary, it promotes the pathological angiogenesis by involving different signalling arm(s)/unknown ligand(s). This review explores the interactions SLIT2/ROBO1, SLIT2/ROBO1–ROBO4, ROBO1/ROBO4, and ROBO4/UNC5B which can be promising and potential therapeutic targets for developmental angiogenesis defects and pathological angiogenesis. Finally we have reviewed the ROBO4 signalling pathways and made an effort to elaborate the insight of this signalling as therapeutic target of pathological angiogenesis.

## 1. SLITs and Roundabouts

The neural and vascular networks often undergo the same routes and similar mechanisms of signalling. Many classes of guidance molecules have been characterized to play critical roles during angiogenesis [1, 2] such as the member of Neuropilin/Semaphorin [3, 4], Ephrin/Eph receptor [5, 6], and Notch/Delta [7–9] gene families. Another class of guidance molecules, SLITs/Roundabouts are increasingly being appreciated [10–12]. SLIT-Roundabout signalling was first identified by studying axonal growth cones [13]. SLITs, the secreted glycoproteins, are the cognate ligand for transmembrane roundabout (ROBO) receptors and induce repulsive signal during axon guidance [14, 15]. SLIT/ROBO signalling axis is also extensively involved in myogenesis [16], kidney induction [17], heart tube formation [18], neuronal and leukocyte migration [19], periodontitis [20], and vascular injury [21]. Recently, this interaction has been shown to be implicated in tumor angiogenesis, where SLITs secreted from cancer cells act as attractants for ROBO1 expressing vascular endothelial cell migration [12]. There are three members of SLIT family (SLIT1, SLIT2, and SLIT3) and four members of ROBO family (ROBO1, ROBO2, ROBO3, and ROBO4). ROBO2 and ROBO3 are highly expressed in the nervous system but untraceable in the vascular system [11]. ROBO1 has been shown to be expressed in both systems. The latest member of this family ROBO4, also called magic roundabout, is a novel endothelial cell specific protein which was identified by using bioinformatic data mining [10]. Spatial expression of ROBO4 also regulated at transcriptional level. Samant et al. [22] have demonstrated that SOX18 mutant mice showed diminished ROBO4 expression in caudal vein endothelial cell compared with wild type mice but showed no change in dorsal aorta endothelial cells. According to earlier views ROBO4 inhibits the migration of heterologous cells (that express ROBO4) and primary endothelial cells by interacting with SLIT2 [11], resulting in downmodulation of tumor growth. Also, studies have demonstrated that both SLITs and ROBOs are extensively expressed in tumors [12, 23–25]. Expression of ROBO4 at the site of neoangiogenesis suggests its involvement in tumor growth [26]. This ambiguous nature of SLIT/ROBO signalling can be explained by the contradictory findings about SLITs that it may either attract [12, 27–30] or repel [11, 31, 32] endothelial cells. Recently, Ballard and Hinck [33] also have reviewed dual role of roundabout receptor in development, epithelial tumor progression, and tumor angiogenesis. In this review we have specially discussed SLIT2-ROBO4 interaction. Among other literatures available regarding switching from chemorepellent to chemoattractant nature of SLIT2, Song et al. [34] have shown that the same guidance molecule may promote attraction or repulsion in neurons in a manner dependent on cAMP activity [34]. They demonstrated that two different functions are possible for a guidance molecule depending on its accompanying molecular physiology. Little is known about the source cells of SLITs. It is possible that exogenous and endogenous SLITs may function differently; for example, the stromal but not epithelial SLITs inhibit vessel growth by downregulating vascular endothelial growth factor receptor (VEGFR) signalling through ROBO4 [35]. Taking another view regarding dual nature of SLIT2, Autiero et al. [36] have suggested that SLIT2/ROBO4 provides repulsive guidance for endothelial cells *in vitro*, whereas SLIT2/ROBO1 shows chemoattractant signalling of endothelial cells *in vivo*. Hence, the current available information is not sufficient to delineate the SLIT2/ROBO1/ROBO4 mediated mechanism of pathological angiogenesis. In this review we have summarized the molecular mechanism of developmental and pathological angiogenesis mediated by magic roundabout (ROBO4) as one of the major processes supporting the tumor growth.

## 2. ROBO4 Has More Distinct Structural Organisation Than Other Family Members

**Figure 1:**
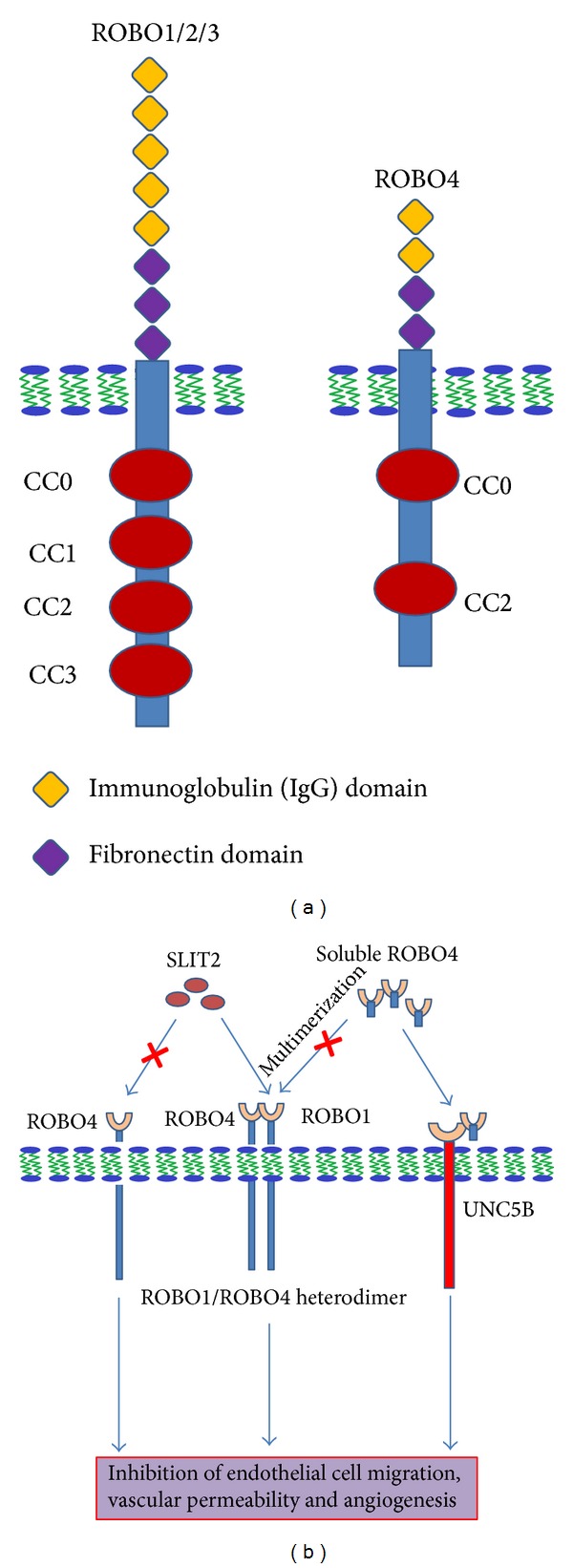
(a) Structural differences among ROBO4 and other ROBOs, (b) Proposed mechanism of soluble ROBO4 to inhibit pathological angiogenesis: SLITs maintain vessel integrity via ROBO1–ROBO4. ROBO1 has more efficient binding with SLIT2 than ROBO4. After SLIT2-ROBO1 binding, deficiency of free SLIT2 triggers the soluble ROBO4 self-multimerization and/or ROBO1–ROBO4 heterodimerization which enhances the SLIT2-ROBO1 signalling promoting the vascular integrity. On the other hand, soluble ROBO4 interacting with UNC5B maintains the vessel integrity by counteracting the VEGF pathway.

Structural organization of magic roundabout shows significant difference with other ROBO members, most notably in the extracellular region (Figure 1(a)). ROBO4 has two immunoglobulin (Ig) domains and two fibronectin domains, whereas other ROBOs have five Ig domains and three fibronectin domains. The cytoplasmic region of ROBO4 has only CC0 and CC2, motifs while ROBO1 contains four conserved CC0–CC3 motifs that are involved in intracellular signalling [37]. Cytoplasmic CC3 domain of ROBO receptor is responsible for interaction with cytoplasmic region of chemokine receptor CXCR4 [38]. Because of the lack of CC3 cytoplasmic domain in ROBO4, it will not be able to interact with cytoplasmic region of CXCR4. It can be speculated that ROBO4 signalling will not be able to regulate the CXCL12/CXCR4 mediated internalization of CXCR4, carcinogenesis, metastasis, and angiogenesis. This structural difference in ligand binding extracellular domain of ROBO1 and ROBO4 may also impart the functional difference. It has been shown that SLIT2-ROBO1 signalling plays an important role in carcinogenesis [39] and attracts endothelial cells during tumor angiogenesis [12]. On the other hand, in many tumors, SLIT2 also has been reported as the putative tumor suppressor being inactivated by promoter hypermethylation [40, 41].

## 3. ROBO4 Inhibits the Pathological Angiogenesis by Maintaining the Vascular Integrity

ROBOs, the neuronal guidance receptors, are newly introduced in pathological vascular development. While ROBO1 is expressed in both endothelial cells and other cell types, ROBO4 is expressed specially in vascular endothelial cells including the tumor vasculature [10, 11, 42]. Considering the guidance molecule, one can speculate that ROBO4 may be repulsive or attractive to migrating endothelial cells during either vascular development. Park et al. [11] have shown that ROBO4 is expressed in primary endothelial cells, interacts with SLIT2 and MENA (known effectors of SLIT-ROBO signalling), and has a role in angiogenesis. Recently, Pircher et al. [43] have used ROBO4 as tumor endothelial marker and demonstrated that increased ROBO4 expression correlated with an increased overall survival in early stage nonsmall-cell lung cancer.

Since ROBOs are extensively involved in guidance mechanism, they should be expressed in the leading end cells. Contrary to this hypothesis, ROBO4 was found to be transcribed in stalk cells (mature vascular cells) of retinal blood vessels and absent from many of the tip cells that sense and respond to extracellular cues. This suggests that ROBO4 may have a biological role that is unrelated to the guidance mechanisms regulating vascular patterning [44]. Park et al. [11] hypothesized that the ROBO4 expression may maintain the phenotype of mature stalk cells by inhibiting processes that are stimulated by proangiogenic factors, such as vascular endothelial growth factor (VEGF). They found that SLIT2 maintains the vessel integrity by inhibiting the vascular endothelial growth factor (VEGF) induced migration, tube formation and vessel permeability *in vitro*, and vascular leak *in vivo*. This happens by blocking the activation of nonreceptor tyrosine kinases (Fyn, Yes, and Src) in ROBO4+/+ endothelial cells but not in ROBO4−/− cells [44]. These results suggest that SLIT2 functions in ROBO4 dependent manner. They have also shown that this SLIT2/ROBO4 signalling axis counteracts the VEGF signalling downstream the VEGFR2 because it had no effect on VEGF induced autophosphorylation of VEGFR2. Src-dependent activation of Rac1 is essential for VEGF induced endothelial cell migration and permeability [45, 46]. SLIT2 treatment of endothelial cells reduces VEGF stimulated phosphorylation of Src family kinases (SFKs), hence preventing Rac1 activation [44] indicating that SLIT2/ROBO4 signalling counteracts the VEGF signalling downstream to VEGFR2 and upstream to SFK. In continuation of these results, it has been shown that ROBO4 mediates the abovementioned mechanism by direct interaction with the intracellular adaptor protein paxillin [47]. ROBO4-paxillin complex formation at cytoplasmic region of ROBO4 blocks activation of GTPase ARF6 consequently blocking Rac [47] and leading to inhibition of VEGF induced endothelial cell migration and vascular permeability [45, 46]. These data strongly suggest that a SLIT2-ROBO4-paxillin network maintains the vascular integrity by inhibiting the VEGF induced neovascularization and vascular leak and therefore may be a novel therapeutic target for diseases involving the vascular system.

Another mechanism, in this context, is the interaction of ROBO4 with UNC5B, a vascular netrin receptor that also counteracts the VEGF signalling [48]. This report demonstrates that the SLIT2 is not the interacting partner of ROBO4, albeit ROBO4 itself acts as a ligand for UNC5B to transmit the inhibitory signal to VEGF induced pathological angiogenesis. Therefore, the interaction of extracellular domain of ROBO4 with extracellular domain of UNC5B may be required to maintain the vessel integrity. This interaction may be between two neighbouring cells (trans) or in a single cell (sis). In Suchting et al.'s [49] experiments, interaction of soluble ROBO4 (extracellular domain of ROBO4) with UNC5B as a ligand may be a possible reason for inhibition of VEGF induced angiogenesis and endothelial cell migration. Treatment with soluble ROBO4 to ROBO4 deficient cells may be a promising agent for diseases characterized by excessive angiogenesis and vascular leak.

**Figure 2:**
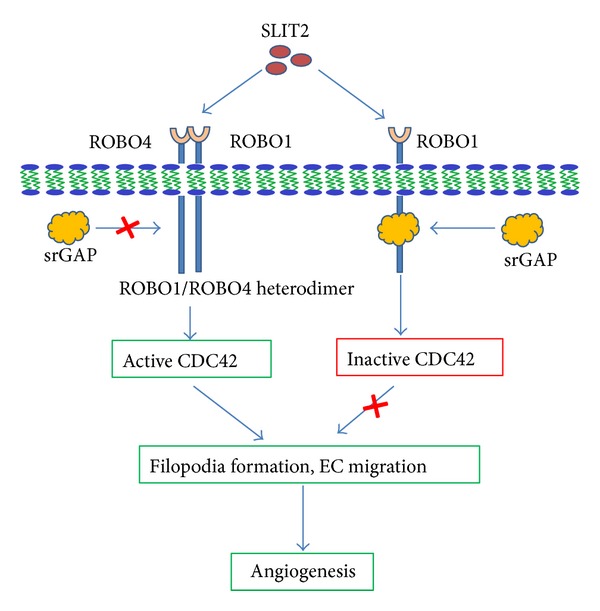
SLIT2 mediated proangiogenic or antiangiogenic activity is dependent on recruitment of srGAP: interaction of SLIT2 with ROBO1 induces the recruitment of Rho GTPase activating proteins (srGAPs) to its CC3 domain which increases the intrinsic GTPase activity of CDC42 leading to reduced level of active CDC42. Reduction of active CDC42 level results in reduced actin polymerization and filopodia formation in endothelial cell. ROBO1–ROBO4 heterodimer formation hinders the binding of srGAP resulting in activation of CDC42, filopodia formation, and ultimate angiogenesis.

**Figure 3:**
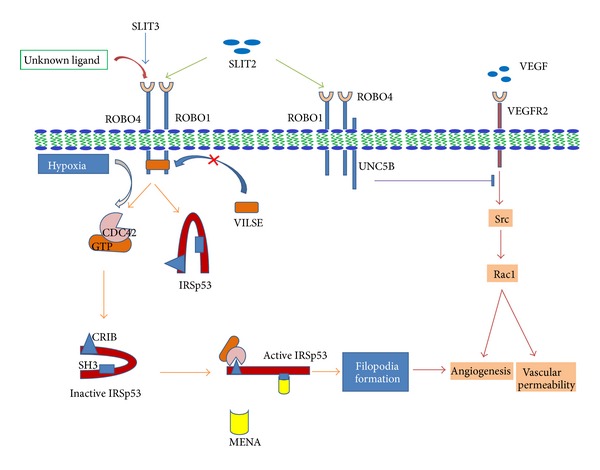
A schematic representation of SLIT/ROBO signalling during angiogenesis**:** SLIT2-ROBO4 signaling inhibits activation of Src and Rac1 to block VEGF driven angiogenesis and vascular permeability, while SLIT2 interacts with ROBO1 and transmits the signal through ROBO1–ROBO4 heterodimer formation. This inhibition is also mediated by binding and signaling through UNC5B where ROBO4 acts as ligand for UNC5B. On the other hand, the heterodimer formation prevents the recruited VILSE to CC2 domain of ROBO4 resulting in increased level of intracellular active CDC42-GTP and IRSp53. CDC42-GTP level can also be increased by hypoxia. The CDC42-GTP binds to CRIB domain of IRSp53 which releases the autoinhibitory effect of IRSp53 and allows the binding of MENA to SH3 domain of IRSp53. MENA recruits the complex to CC2 domain of ROBO4's cytoplasmic region or directly mediates actin nucleation resulting in filopodia formation and directional migration promoting the angiogenesis.

The N-terminal Ig like domains of ROBO1 and ROBO4 are 42% identical but the residues identified for SLIT2 binding are not conserved in ROBO4 and are replaced by residues that are not compatible with binding of SLIT2. This suggests that ROBO1 has more efficient binding with SLIT2 compared to ROBO4. Park et al. [11] have demonstrated that application of SLIT protein inhibits endothelial cell migration and Jones et al. [44] show that SLIT2 does not respond in ROBO4 deficient mice, indicating that SLIT2 acts in a ROBO4 dependent manner [44]. Contrary to this, addition of soluble ROBO4 is able to inhibit angiogenesis, endothelial cell migration, and growth but unable to interact with any known SLIT protein while ROBO1 interacts with all three known SLITs [49]. Recently it also has been demonstrated that secreted SLIT3 guides vascular development by directing ROBO4-positive endothelial cell movements [50, 51] (Figure 3). This interaction still needs to be established. Probably SLITs signal by interacting with ROBO1 and soluble ROBO4 inhibits this signalling by interacting with ROBO1 [49, 52]. This explanation is further supported by a report demonstrating that ROBO1 and ROBO4 interact and share molecules such as SLIT2, MENA, and VILSE, CDC42-GTPase-activating proteins (CDC42-GAP) [24]. Possible speculations are (1) SLITs probably function by interacting with ROBO4, and because of multimerization of soluble ROBO4 [53], it was not able to interact with SLIT in Suchting's experiment (Figure 1(b)) or (2) SLIT2 might mediate its function via ROBO4 by indirect binding with other receptors, such as ROBO1 or Syndecans [54] (Figure 1(b)). ROBO4 has been coimmunoprecipitated with ROBO1 in cultured endothelial cells *in vitro *[29, 32] suggesting that ROBO1–ROBO4 heterodimerization can be the mediator of ROBO4 responsiveness to SLIT2 [10, 32, 55]. In another recent report interaction of immobilised SLIT2 was also inhibited by anti-ROBO1, anti-ROBO4 blocking antibody suggesting the implications of both of the receptors [56]. Therefore, we hypothesize that SLITs maintain vessel integrity via SLIT2/ROBO1–ROBO4 interaction and because of more efficient binding of SLIT2 with ROBO1, it preferentially binds to ROBO1, leading to the deficiency of free SLIT2. This SLIT2 deficiency triggers the soluble ROBO4 self-multimerization and/or ROBO1–ROBO4 heterodimerization [53] which enhances the SLIT2/ROBO1–ROBO4 signaling promoting the vascular integrity (Figure 1(b)).

Hence, there are three complexes that can be exploited to target the pathological angiogenesis: SLIT2/ROBO1, ROBO4/UNC5B, and ROBO1/ROBO4. By promoting or inhibiting the affinity and/or association of these axes according to the mechanism involved in a particular system, inhibition of pathological angiogenesis and vascular high permeability can be appreciated as therapeutic target.

## 4. ROBO4 Also Promotes Angiogenesis via Different Signalling Arm or Unknown Ligand

We discussed that ROBO4 has endothelial specific expression and SLIT2/ROBO1–ROBO4 signalling inhibits the endothelial cell migration and pathological angiogenesis by counteracting the VEGF signalling. Contrary to this antiangiogenic nature of ROBO4 signalling, there is also evidence supporting that ROBO4 promotes the pathological angiogenesis [27, 57]. Binding of SLIT2 to ROBO1/4 complex communicates a signal through the actin cytoskeleton to induce filopodia formation leading to endothelial cell migration, a fundamental event of angiogenesis [29]. This also has been supported by knockdown and overexpression approaches in zebrafish (*Danio rerio*) demonstrating that ROBO4 plays an essential role during angiogenesis and guides endothelial cells to their target analogous to ROBO1, ROBO2, and ROBO3 during neuronal guidance [57]. SLIT2 independent ROBO4–ROBO4 dimerization through the cytoplasmic domains provides directional guidance for sprouting, and loss of ROBO4 results in sprouting in wrong direction eventually leading to regression [57]. To explain this proangiogenesis nature, it has been proposed that ROBO4 activates Rho GTPase CDC42, Rac in endothelial cells, leading to the formation of filopodia and lamellipodia. Filopodia formation is thought to be involved in sensation of chemotropic cues and directed migration of cells. Angioblasts (extraembryonic mesenchyme cells that differentiate into endothelium) isolated from the ROBO4 deficient embryos show behavior characteristic of cells searching for guidance and show lower amounts of active CDC42 in lysates [27]. Collectively, these results suggest a role of proangiogenic mechanisms for ROBO4 in vascular guidance. Further, by using two mutants of ROBO4 (c-ROBO4, lacking partial N-terminal and C1-ROBO4, and lacking the N-terminus completely), Kaur et al. [27] demonstrated that both intracellular and extracellular domains of ROBO4 are involved in the activation of CDC42. They argue that ligand dependent recruitment of guanine nucleotide exchange factors (GEFs) activates CDC42/Rac and recruitment of GAPs inactivates CDC42/Rac [27].

Evidence shows that SLIT2 is not the ligand for ROBO4 because (1) N-terminal ligand binding domain of ROBO4 is not fit for SLIT2 binding; (2) dimerization of cytoplasmic region of ROBO4 is independent of SLIT2 binding [57]; (3) ROBO4 activates the Rho GTPase in endothelial cell [27], but SLIT2 mediated inhibition of endothelial cells migration does not show change in level of active Rho GTPases [32]. Kaur et al. [27] have shown that both mutants of ROBO4, either lacking complete N-terminus or C-terminus, are unable to activate the Rho GTPase. These results indicate that ROBO4 mediated activation of Rho GTPases is independent of SLIT2-ROBO4, interaction but it is ligand dependent. Recently, it was demonstrated that SLIT2-ROBO1 interaction suppresses the activity of CDC42 [58]. This may be an indication towards the involvement of SLIT2/ROBO1-ROBO4 interaction in regulation of the activation of Rho GTPases. Another similar report shows that hypoxia exhibits activated CDC42, Rac1 protein expression which induces hypoxia-inducible factor-1*α* (HIF-1*α*) leading to VEGF production and angiogenesis [59]. Also, it has been demonstrated that hypoxia significantly increased both mRNA and protein levels of SLIT2, ROBO1, and ROBO4 in HUVEC [60]. It can be speculated that hypoxia induced activation of CDC42 and Rac1 proteins and endothelial cell migration leading to angiogenesis may be mediated via ROBO1 and ROBO4. The above discussion concludes that ROBO4 mediates the ligand dependent activation of Rho GTPase regulating the angiogenesis, but this regulation may not be dependent on SLIT2-ROBO4 interaction.

In neurons interaction of SLIT2 with ROBO1 induces the recruitment of Rho GTPase activating proteins (srGAPs) to its CC3 domain which increases the intrinsic GTPase activity of CDC42 leading to its inactivation [61] (Figure 2). Localized deactivation of CDC42 near the SLIT source results in asymmetric actin polymerization that induces the cells to migrate away from the source of SLIT, leading to repulsive guidance signalling [62–64]. In endothelial cells, knockdown of ROBO4 abrogated the chemoattractive response to serum but enhanced chemoattractive response to SLIT2 [32] supporting the hypothesis, that other than SLIT, an unknown ligand may bind ROBO4 leading to activation of CDC42 and attraction guidance signalling (Figure 3).

How does ROBO4 mediate the proangiogenic attraction guidance signalling in endothelial cell? Again as discussed earlier there could be two possibilities: (1) an unknown ligand for ROBO4 and (2) SLIT2 may signal by interacting with ROBO1–ROBO4 heterodimer. There should be a protein interacting with cytoplasmic region of ROBOs, to determine the antiangiogenic or proangiogenic nature of this signalling. One such appreciable protein is srGAP (SLIT-ROBO GTPase activating protein). These proteins are particularly abundant in SLIT responsive regions. Binding of srGAP to CC3 domain of ROBO1 increases the intrinsic GTPase activity of CDC42, which converts the GTP-CDC42 into GDP-CDC42 inactivating CDC42. Reduction of active CDC42 eventually decreases actin polymerization affecting the cell movement [61]. Repulsion mechanism of SLIT can therefore be explained by the relative amount of actin polymerization on the side of the cell proximal and distal to the SLIT source. Proximal side having relatively less actin polymerization than the distal side results in the movement of cell away from the SLIT source. To explain the question raised in the beginning of this paragraph, we hypothesize that ROBO1–ROBO4 heterodimerization may prevent recruitment of srGAPs to CC3 domain of ROBO1 resulting in activation of CDC42 leading to attraction guidance signalling (Figure 2). So, the possibility that ROBO1–ROBO4 dimerization facilitates ROBO4 signalling downstream of SLIT2 without direct interaction between ROBO4 and SLIT2 cannot be denied. However, it is still unclear whether srGAPs naturally bind to ROBO1 only or to ROBO1–ROBO4 heterodimer. If they bind to ROBO1 only, after overexpression of ROBO4, the heterodimer formation will prevent the interaction of srGAPs with ROBO1 resulting in increased level of active CDC42 leading to lamelipodia and filopodia formation in endothelial cell. If they bind to heterodimer leading to inactivation of CDC42, then the overexpression of ROBO4 will lead to inactivation of CDC42, so in this case probability of an unknown ligand for ROBO4 remains open. VILSE, a CDC42-GAP, was shown to interact with ROBO4 resulting in SLIT2 dependent increased level of active CDC42 [32]. Similar to abovementioned srGAP mediated ROBO signalling (Figure 2), this VILSE mediated activation of CDC42 is possible only if ROBO1–ROBO4 heterodimer formation prevents the binding of VILSE to ROBO4. Our discussion converges towards a mechanism where, either SLIT2 mediate the GTPase mediated angiogenesis via SLIT2/ROBO1–ROBO4 signalling or an unknown ligand other than SLIT2 is playing a role.

To understand the mechanism of ROBO4 to promote or inhibit the fundamental events of angiogenesis, it is important to explore the other interacting partner protein(s) with intracellular region of ROBO4, especially proteins involved in actin regulatory machinery. The novel proteins interacting with ROBO4 involved in actin regulatory machinery include the Wiskott-Aldrich syndrome protein (WASP) and neuronal WASP proteins (NWASP) and their regulatory proteins: WASP-interacting protein **(**WIP) and syndapin [65]. WASP has been previously implicated in ROBO4 mediated migration of HEK-293T cells [27]. Glutathione S-transferase pulldown experiments demonstrate the interaction of MENA, WASP, and NWASP with the intracellular domain of ROBO4, and a proline-rich domain in NWASP is required for binding to intracellular domain of ROBO4 [29]. The identification of WIP as an interacting partner of ROBO4 is interesting because ROBO4 may activate WASP-dependent actin polymerization through interactions with WIP. NWASP-syndapin interaction may be required for generation of ROBO4-labeled vesicles and endocytosis [66]. Thus, ROBO4 acts as a molecular scaffold to recruit these actin nucleation proteins to enable the actin assembly and filopodia formation.

It is obvious that interaction of activated CDC42 (CDC42-GTP) presumably with other proteins is needed for the recruitment of MENA, WASP, NWASP, and WIP to ROBO4 for the formation of filopodia and migration. One such protein previously implicated in CDC42-GTP mediated filopodia formation is an SH3 domain-containing insulin receptor substrate protein 53 (IRSp53) (Figure 3). IRSp53 protein interacts with CDC42-GTP via CRIB motif of IRSp53. The interaction of CDC42 with CRIB motif relieves an intramolecular autoinhibitory effect of IRSp53 allowing the recruitment of MENA to the SH3 domain of IRSp53 [67]. MENA mediates actin nucleation resulting in filopodia formation and directional migration by recruiting CDC42-IRSp53-MENA complex to CC2 domain of the cytoplasmic tail of ROBO4 (Figure 3). So the above discussion indicates that ROBO4 promotes the lamellipoda, filopodia formation in endothelial cells leading to angiogenesis via interacting either with SLIT2/ROBO1 complex or with an unclassified ligand.

## 5. Question to Be Addressed 

Our discussion converges to the point that ROBO4 is a key mediator of pathological angiogenesis and unravelling its mechanism of action may prove it as double edged sword. To unravel its mechanism there are some key questions to be addressed such as the following: (1) What are the physiological partners of ROBO4? (2) Does the choice of partner proteins of ROBO4 depend on microenvironment of cell that determines the proangiogenic or antiangiogenic nature of ROBO4 signalling? (3) What are the factors that regulate the homodimerization or heterodimerization of ROBOs? (4) Syndecans, a conserved family of heparan- and chondroitin-sulfate, are emerging as central players in cell surface interactions. It has been demonstrated that heparan sulfate serves as essential co-receptor in Slit-Robo signalling [68, 69]. Does heparin-sulfate contribute to the ambiguous nature of ROBO4 signalling in angiogenesis?

## 6. Conclusion

Converging points of evidence discussed in this review indicate that ROBO4 has prominent role in regulation of angiogenesis. ROBO4 inhibits the pathological angiogenesis by counteracting the VEGF signalling either via SLIT2 or by interacting with UNC5B. This inhibition mechanism may be dependent on SLIT2-ROBO1 and ROBO1–ROBO4 interactions. ROBO4 signalling also promotes the pathological angiogenesis either via SLIT2/ROBO1–ROBO4 axis or via interaction of ROBO4 with an unknown ligand other than SLIT2. SLIT2 signalling via ROBO1–ROBO4 heterodimerization and srGAP interaction with cytoplasmic region of ROBO1 may be responsible for proangiogenic attraction guidance signalling in endothelial cells. Most of the literature considering therapeutic aspect of ROBO4 is focused on interaction of SLIT2 and ROBO4. In this review we have attempted to explore whether ROBO1–ROBO4 interaction can be targeted for antitumorigenic and antiangiogenic therapy. Since ROBO4 has dual behaviour of promoting and inhibiting angiogenesis depending on cell/tissue type, the therapeutic agents having properties of promoting and inhibiting ROBO4 signalling, respectively, can be used to target tumor angiogenesis according to the cell/tissue type under consideration. Because of its specific expression at the site of neoangiogenesis, ROBO4 can be used as angiogenesis marker. Although many questions still remain to be addressed regarding ROBO4 signalling, its understanding may be a milestone in targeting the pathological angiogenesis.
